# A research utilisation framework for informing global health and development policies and programmes

**DOI:** 10.1186/s12961-018-0284-2

**Published:** 2018-02-09

**Authors:** Christine Kim, Rose Wilcher, Tricia Petruney, Kirsten Krueger, Leigh Wynne, Trinity Zan

**Affiliations:** 1 FHI 360, Durham, NC United States of America; 2 Gillings School of Global Public Health, Health Policy and Management, University of North Carolina, Chapel Hill, NC United States of America

**Keywords:** Research utilisation, Evidence-to-practice, Knowledge translation, Knowledge brokers, Research uptake, Evidence-informed policy, Research utilisation framework, Research utilisation case study

## Abstract

A shift in the culture and practice of health and development research is required to maximise the real-world use of evidence by non-academic or non-research-oriented audiences. Many frameworks have been developed to guide and measure the research utilisation process, yet none have been widely applied. Some frameworks are simplified to an unrealistic linear representation while others are rendered overly complex and unusable in an attempt to capture all aspects of the research utilisation process. Additionally, many research utilisation frameworks have focused on the policy development process or within a clinical setting, with less application of the translation process at the programme level. In response to this gap – and drawing from over a decade of experience implementing research utilisation strategies – we developed a simple, four-phase framework to guide global health and development efforts that seek to apply evidence to policies and programmes. We present a detailed description of each phase in our framework, with examples of its relevance and application illustrated through our own case study experiences in global health. We believe the utility of this framework extends beyond the health sector and is relevant for maximising use of evidence to achieve the Sustainable Development Goals.

## Background

In 2004, WHO published the World Report on Knowledge for Better Health [1], emphasising the need for greater investments in translating knowledge into action to more quickly foster country-level evidence-informed policies and for implementation and scale-up of proven life-saving interventions. While general agreement exists that these improvements are needed, researchers and policy-makers have identified many barriers and challenges that inhibit the research utilisation process [1–5]. A shift in the culture and practice of health and development research is required to maximise the real-world use of evidence by non-academic or non-research oriented audiences. Despite the many calls for greater action to bridge the ‘know–do gap’ or to develop evidence-informed policies, affecting change in the culture and practice of research is slow and requires deliberately managed and sustained efforts [1, 6, 7].

In response to the know–do gap, publications on the conceptualisation of research utilisation (RU) – a term that is often used interchangeably with knowledge translation, research uptake or evidence-informed policy – have increased, and included identification of barriers and facilitators to use as well as strategies to improve research uptake [2, 3, 5, 8]. Consequently, many frameworks have been developed to guide and measure the RU process, but few have been widely applied, in part because the dynamic processes and actors involved are diverse and context-specific, but also because some frameworks provide limited ‘how-to’ support for implementation [9–11]. Some frameworks represent RU within a linear process [12], while others present a more complex RU process, capturing the dynamic relationships between research producers, end users of research, and ‘intermediaries’ [9, 10, 12] to target the best RU output balancing end users’ ‘knowledge need’ and appropriate ‘evidence response’ [13]. Despite their attempts to represent the dynamic nature of RU, many of the existing RU frameworks have been criticised as inadequate due to their lack of direct links between research and its use [4] or because of their inapplicability to low- and middle-income country (LMIC) contexts and cultures [4, 8, 10]. A scoping review of knowledge brokering found that guiding conceptual frameworks and models varied, focused on linkage and exchange, and depended on multiple frameworks for guidance, yet strong evidence for effective knowledge brokering approaches were limited [14]. Another recent scoping review of integrated knowledge translation strategies found that there is a lack of explicit description of underlying logic or use of frameworks for associated strategies or approaches implemented for increasing research uptake [15].

Additionally, published RU frameworks have focused largely on the policy development process or within a clinical setting, with less application of the translation process at the programme level [5, 16–19]. The application of evidence at the programme level is particularly salient for supporting the replication, scale-up and institutionalisation of evidence-based programmes and practices within a country. While the impact of evidence-based programming has been demonstrated through research, descriptions of specific strategies to translate and adapt evidence-based programmes to other contexts remains sparse, particularly in LMICs [1, 4, 6, 8].

In response to the need for a RU framework driven by both policy and programme needs, inclusive of specific RU strategies and applicable to LMICs, we developed a simple, four-phase framework that builds on the current RU literature and reflects over a decade of experience implementing RU strategies to improve global health policy and practice.

## A RU framework

**Fig. 1:**
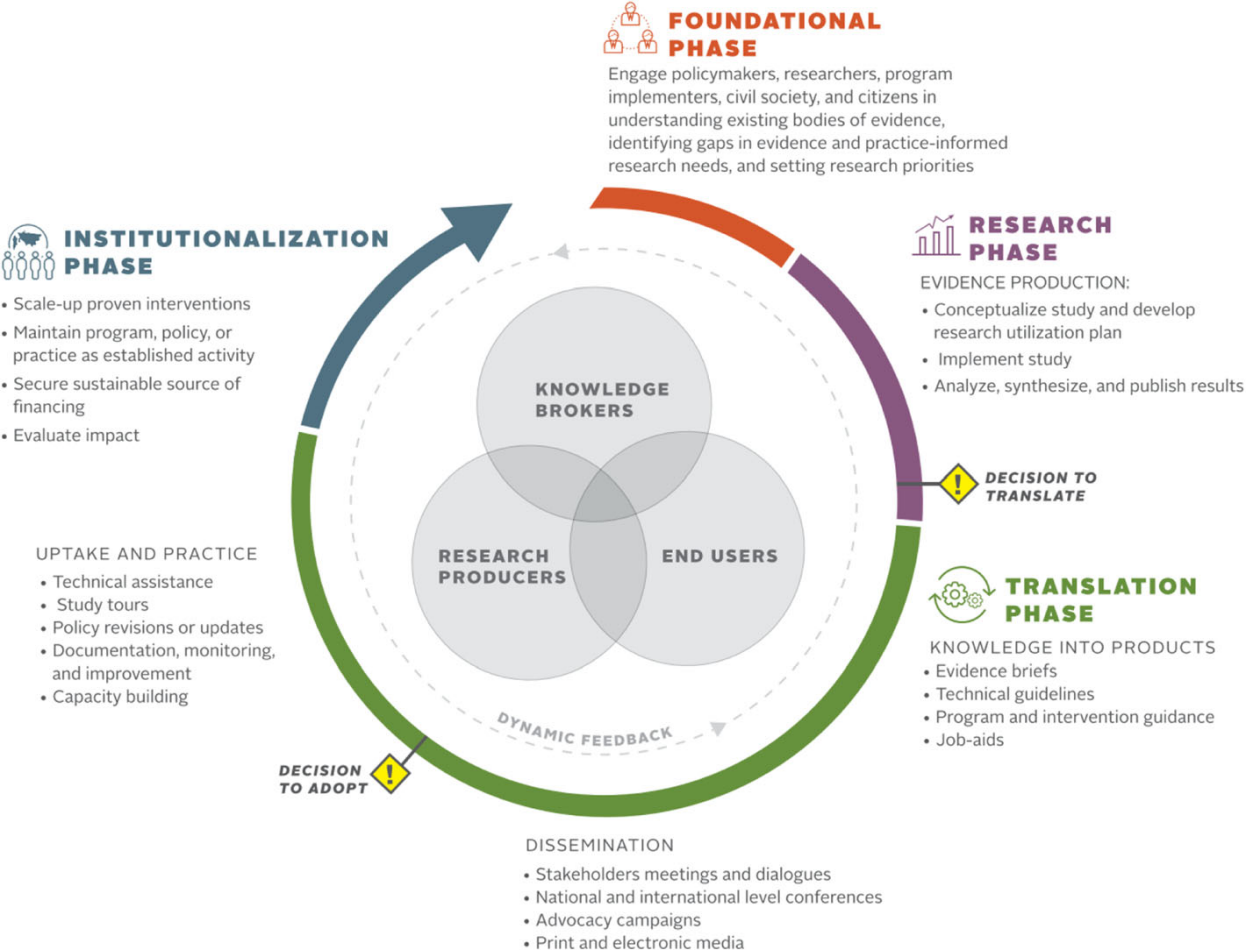
FHI 360 Research Utilisation Framework

Figure 1 shows the FHI 360^1^ RU framework, which consists of four phases, namely foundation, research, translation and institutionalisation. Three key types of actors – evidence producers, knowledge brokers and end users of evidence – are essential to the progress and flow of each phase and, though these groups are categorised distinctly, they often overlap and are not mutually exclusive. Two crucial decision points for translation and adoption are identified at the end of the research phase and during the translation phase, respectively [20]. The circular representation of the model demonstrates the dynamic nature of the process; actors may stop at any point along the continuum and return to a prior phase. Furthermore, experience demonstrates that activities in different phases may be implemented concurrently, overlapping, or back and forth. This dynamic process allows for constant learning and evaluation within and between phases. The circular representation also captures the importance of policy, programmes and practice feeding back into research. The framework was inspired by, and adapted in part, from two existing frameworks by Wilson et al. [16] and El Jardali et al. [20], both of which built on existing knowledge translation theories and models. As a long standing RU programme in global health, we often referred to the Wilson et al. framework [16], although we found it to have similar limitations as reported above. A product of the Centers for Disease Control and Prevention (CDC), the framework emphasised clinical evidence, did not identify key actors, and did not have a foundational phase. The El Jardali et al. [20] framework was referred to for its policy application, yet remained limited for our programmatic needs, as there was less emphasis on programme implementation compared to policy implementation. The combination of some key concepts of these frameworks, in addition to our own experiences in the field, led us to develop a simplified framework to inform programmes and policies in LMICs. The simplicity of the framework is important from a programmatic perspective as it emphasises key areas within a very complicated process, making it more digestible for a broader audience. Central to our framework is the engagement and interaction of key actors essential to the RU process. This human element distinguishes the framework from others as it drives the context and climate for RU – the relationships, capabilities, politics and demand for evidence – that determine the success of evidence uptake. In order to keep the framework simple, we have excluded some elements that we felt were already obvious such as larger contextual factors. While many external factors may dictate the success or failure of research and evidence uptake, such as capacity or political climate, they are not visually captured.

We present a description of each phase in our framework and provide examples of its relevance and application within global health research, policy and programming. Although we focus on our experiences in global health, we believe the utility of this framework extends beyond the health sector and is relevant for concerted action to achieve the Sustainable Development Goals.

### Key actors in RU

The three categories of key actors represented in the framework are evidence producers, knowledge brokers and end users of evidence. These key actors have differing roles and commitments throughout each of the phases. Individual evidence producers and knowledge brokers are typically from academic institutions and research or policy institutes such as think tanks, or are employed within research units of governmental or non-governmental organisations. Organisations and institutions may also be considered evidence producers and knowledge brokers, in addition to individuals. Evidence producers, as the name suggests, are those who lead the design and execution of research or data collection activities. Knowledge brokers are considered intermediaries to help communicate evidence and facilitate evidence use between producers and end users [14]. End users are those expected to apply evidence to programmes or policies and can range from policy-makers to advocates, programme implementers, members of the media and civil society organisations.

The capacity of individuals or institutions to perform these three key roles within a country may differ. For instance, the capacity of local evidence producers to supply high-quality research and data may be limited in some countries as compared to others; this is often the case in LMICs, though many donors have been directing resources for years toward building local research capacity [21, 22]. Additionally, capacity to use and apply evidence among end users may be a constraint to achieving evidence-based policies and programmes in some countries despite donor-funded projects that have also focused on building capacity of end-users to seek out, understand and apply evidence [23]. Even when capacity exists in a country both to produce and to apply evidence, research uptake is not always achieved because evidence-producers and end-users are not linked to each other [24]. Furthermore, the use of evidence can be of a political nature as it is linked to the human element and how people make decisions based on relationships and political dynamics. Thus, knowledge brokers are the important third category to help bridge the gap between producers and end users.

### Foundation phase

The foundation phase emphasises the engagement of various stakeholders to understand the most pressing knowledge needs and to set research priorities. Stakeholders who are involved in research priority-setting may include policy-makers, civil society representatives, researchers, donors, health providers, private sector representatives, advocates, implementing partner organisations and individual citizens. The primary aim of research priority-setting is “*to gain consensus about areas where increased research effort including collaboration, coordination, and investment will have wide benefits to society*” [25]. The foundation phase guides the overall process and is instrumental to establishing on-going relationships and collaborations throughout the rest of the phases. In practice, stakeholder engagement occurs at all points in the framework and throughout a study, but may not be initiated from the beginning of a study. However, the framework emphasises that, ideally, engagement activities should happen at the start, building on experiences of successful evidence uptake that is often based on the strength of relationships and engagement with key stakeholders.

Research priority-setting is particularly important in contexts with limited financial and human resources as it is essential to maximise the impact of research investments [26]. However, approaches for engaging diverse stakeholder groups in evidence-based research priority-setting in LMICs have not been evaluated or documented well. Nonetheless, the value and function of research priority-setting is well-recognised and instrumental in strengthening country-level research capacities overall, a process that lays the foundation for greater use of investments in conducting needed research and applying findings [1, 27]. Research priority-setting can be a complex activity; a checklist outlining nine common themes of good practice for research priority-setting was developed by Viergever et al. [26] to assist priority-setting processes by countries.

Priority-setting at the country level should be country-led and feed into national strategies and implementation plans. Specific activities related to research priority-setting can include open fora, town hall meetings, conferences, targeted consultations or workshops. These activities allow different stakeholder groups to interact and identify synergies for achieving shared goals and objectives, within or across sectors.

### Research phase

The research phase consists of study conceptualisation and implementation, including protocol development, data collection, and data analysis and interpretation. While researchers are the main actors during implementation and analysis within our framework, all key actors – evidence producers, knowledge brokers and end users – take part in study conceptualisation to ensure its relevance, which maximises eventual uptake of the findings. FHI 360 developed a RU planning tool, most often applied by the RU expert playing the knowledge broker role, as a guide to consider determinants of eventual uptake and to make decisions regarding research design, stakeholder engagement and communication strategies, and dissemination approaches. For instance, a key recommendation for intervention studies or pilot feasibility projects is to design the intervention as part of the existing system, rather than rolling out a highly complex and managed model, in order to increase the likelihood that it can be scaled up [28]. Another recommendation is to document the implementation process for the intervention in order to identify best practices and avoid pitfalls for future replication [29]. We have developed and used an intervention tracking tool to help document the implementation process. The tool aims to pinpoint when and how the intervention differed from the original design, identify activities which are not always included in final research reports and, most importantly, articulate what is needed for scale-up if the pilot is successful.

Engagement of stakeholders throughout the research phase, not only during conceptualisation but also during the development of a research utilisation plan, data analysis and interpretation, and results synthesis, supports greater participation during dissemination and uptake of recommendations [28]. Stakeholder analysis can be used to help identify key stakeholders and how to critically engage them throughout the RU framework phases. Several tools exist to facilitate stakeholder identification and analysis, including one that we developed as part of our package of RU tools.^2^

At the end of the research phase, the framework notes a critical decision point – the decision to translate. The decision to translate is the active decision to develop and disseminate knowledge products based on research findings to drive thoughtful discussion and widespread use of the evidenced-based programme, practice or policy [20]. Ideally, this is a collective decision taken by all three categories of key actors and helps bridge the research to translation phases. Importantly, we characterise this as a ‘decision point’ because uptake of even high quality, compelling evidence is not automatic and requires deliberate action. While the framework includes a research phase, non-research field practitioners may find the framework applicable at this decision point – to move an existing body of evidence or programme practice to inform policy and other practice.

### Translation phase

The translation phase actualises the first decision to start turning evidence into actionable products for wide dissemination and use. Through its three sub-phases and a decision point, knowledge products are created, dissemination and advocacy activities are conducted to promote wide adoption of the evidence, and uptake of evidence-based practices or research results is actively supported. This phase is the longest in the framework, signifying that it often takes time to achieve uptake and transition to true institutionalisation.

According to the framework, evidence producers and knowledge brokers work together to develop knowledge products that effectively synthesise evidence and facilitate its application to programmes and policies. Knowledge products may include evidence briefs, technical guidelines, programme and intervention guidance, and job aids. Studies with decision-makers have shown that key messages, or take-home messages, that are solution-oriented and in short form, such as evidence briefs, are more effective than a research report [30]. Targeted messages to the audience of interest are also important for an effective knowledge-transfer strategy. Thus, multiple audience-specific messages should be generated – for decision-makers as well as programme users – in a way that adapts to the language of the intended users [31–33]. Users of research who are intended to implement the new policy or programme require additional products, such as technical guidelines and job aids, which can help to ensure that practitioners are changing behaviour and routinely applying evidence-based practices in their jobs. Audiences intended to use job aids or other knowledge products should be engaged in their development and field-testing [28, 34].

A dissemination strategy for spreading research results or evidence-based practices to target audiences through the most effective channels [28, 35], which was developed as part of the research utilisation plan during the research phase, is also implemented during this phase. Evidence-based knowledge products can be disseminated through forums, policy dialogues, national and international conferences, advocacy campaigns and media engagement. While the mechanism and forum for dissemination is important, the voice carrying the message is equally as important as the messages themselves. Identifying and engaging champions or an opinion leader to take an active role in dissemination activities can increase the likelihood for uptake of an evidence-based practice or programme [35, 36]. The target audiences identified in the dissemination plan will inform what messages and avenues for dissemination are most effective and strategic for the greatest uptake.

The decision to adopt the evidence-based programme, practice or policy is the second critical decision point along the framework and is made by end users, including communities, programme managers, implementing organisations or policy-makers [20]. End users may require additional evidence or have other questions that require investigation resulting in a feedback loop to the research phase before the decision to adopt can be made.

Once end users have decided to adopt a piece of evidence into policy and/or programmes, it must be applied in real-life scenarios. To do so, evidence-producers and knowledge brokers may provide technical assistance to end-users to conduct study tours to see application of the evidence at scale, revise or update policies or norms, develop monitoring and improvement plans, and/or build technical capacity for implementation. Adoption and replication of an effective intervention or practice often requires the proper policies and guidelines that are relevant to the local context to be in place, testing and refining of the core programme elements, and training practitioners to implement appropriately, with ongoing assistance and capacity-building [29, 37, 38]. Documentation, monitoring and evaluation of these processes, including client-level outcomes, as well as identifying which populations benefit more or less, are essential to building more evidence for the practice in other contexts. Often, programmes are introduced without readily assessing the environment for adequate policies or readiness for introduction of new interventions. A more strategic approach to uptake and practice should lead to more sustainable programming in the future.

It is important to note that a given RU effort may jump between or move concurrently through the subcomponents of the Translation phase, or between and through the Translation and Institutionalisation phases, as happened in our case study of community-based family planning (CBFP) in Zambia described below.

### Institutionalisation phase

Institutionalisation of evidence-based programmes, practices and policies is the main outcome of the evidence translation process. The institutionalisation phase is where evidence use is established as an organisational norm and proven interventions are maintained, funded and scaled-up [20]. Sustainability of health and development programmes is determined by a range of factors, including the availability of human and financial resources, integration into larger systems and institutions, and ongoing political will and support, and requires continuous and systematic efforts over long periods of time in order to ensure that evidence is truly institutionalised [39].

Identifying sustainable human resources and financing mechanisms are the biggest challenges in the institutionalisation of promising and innovative solutions in low-income countries. While low-income countries take on over 50% of the global disease burden, they account for less than 2% of global public health spending [40]. Efforts to create a more coordinated global architecture for development targets and financing have been made through the Millennium Development Goals and its follow-on Sustainable Development Goals. Successfully achieving these goals will require greater coordination and alignment by country stakeholders and international partners [41] to agree upon evidence-based interventions as well as the strategies and resources to implement and scale them. Though not always the case, in our experience that we present from Zambia, consistent donor-support (in this case, from the United States Agency for International Development (USAID)) aligned to global consensus for a particular practice (WHO endorsement of community-based provision of injectables) helped local stakeholders move from introduction to institutionalisation. Sustainable financing and human resources ensure that evidence-based programmes and services are not disrupted and can therefore result in greater development and health impacts. The availability of well-trained human resources from field implementers to policy-makers sets the stage for institutionalisation through systematic changes in norms and practices.

## Zambia case study

### Background

Herein, we will describe a RU framework experience from a demonstration project of a CBFP programme in which depot-medroxyprogesterone acetate (DMPA) was added to the family planning (FP) methods already being provided by community-based distributors (CBD) in Zambia [42]. In Zambia, an inequitable distribution of FP service use exists among rural women compared to urban women due to traditional misconceptions and myths, long distances to health facilities, frequent stock-outs of contraceptives at both health facility and community levels, and inadequately trained personnel resulting in long queues at health facilities. CBDs have historically played an important role in providing CBFP services to women in rural areas, though their impact is often limited by providing only condoms and oral contraceptive pills. Delivery of DMPA, one of the most popular methods among women of reproductive age, by CBDs, would help expand the reach of FP services and meet the reproductive health needs of women in rural areas. Studies in other countries found this delivery approach to be safe and effective. The pilot study in Zambia aimed to examine the incremental or additive effect of CBDs providing DMPA, including (1) their ability to provide DMPA to clients safely and effectively; (2) the acceptability of, and client satisfaction with, CBD agent delivery of DMPA; (3) if and how the workload of CBD agents and their supervisors changed with the addition of CBD provision of DMPA; and (4) the additional cost per couple-years of protection of adding DMPA to the existing CBD-delivered FP programme. The study was implemented by FHI 360 and ChildFund Zambia, in coordination with the Zambia Ministry of Health (MOH) and with support from the USAID.

### Key actors in the pilot study

There were several key actors involved in the CBFP demonstration project in Zambia. End users were comprised of the Government of Zambia MOH officials, health workers and associations of health professionals, and non-governmental organisations implementing community health programmes in the country. Evidence producers were researchers at FHI 360, who led the research and supported implementation of the programme by Childfund Zambia. ChildFund staff were both evidence producers, as they were deeply involved in study design and data interpretation, and end users since, as they were able to incorporate findings into future programme implementation. Knowledge brokers included RU experts within FHI 360 who partnered with researchers and worked closely with implementers and end users throughout and after the study. The building, re-building and strengthening of relationships among key actors was instrumental in the institutionalisation of the programme, particularly as unforeseen changes in the government structures and policy-level stakeholders occurred during the process.

### Foundation phase: growing demand for a CBFP research agenda

The 2006–2010 National Health Strategic Plan of Zambia identified “*strengthen family planning and contraceptive choice programmes, with a special focus on rural districts*” [43] as a key strategy for decreasing maternal mortality and increasing access to integrated reproductive health. Globally, WHO began advocating for increased task sharing of some health services to lower cadres in order to rapidly increase healthcare access needs and address human resource crises in underserved areas [44, 45]. In 2008, discussions in Zambia began regarding the inclusion of DMPA into the CBD programme and, by 2009, the MOH requested that pilot research be conducted on the delivery of DMPA by CBD agents.

### Research phase: implementation of the pilot study

With approval from the Government of Zambia, FHI 360 and ChildFund convened an initial stakeholder meeting to discuss the design of the pilot study to add injectable contraception to ChildFund’s CBFP programme. Government officials and national stakeholders requested measures of programme impact to be included, in addition to local confirmation of the safety, feasibility and acceptability of CBD agent provision of injectable contraception. From November 2009 to February 2011, a RU expert, acting as knowledge broker, supported the study during this phase, managing stakeholder engagement, contributing to study design, tracking cost information and documenting implementation details using the intervention tracking tool. Preliminary findings were presented to a small group of stakeholders in May 2011. At this meeting, a ‘decision to translate’ effectively occurred when the FP Technical Working Group made several recommendations to the MOH based on the preliminary findings. These included to continue provision of DMPA by CBD agents in pilot districts, develop a phased national scale-up plan, and revise policy to permit CBD agents to administer DMPA. During this time, a high level well-respected champion was brought on board to present at the meeting and help implement recommendations, greatly facilitating the transition to the translation phase.

### Translation and institutionalisation: a winding path to wide-spread and government-supported CBD of DMPA

**Fig. 2:**
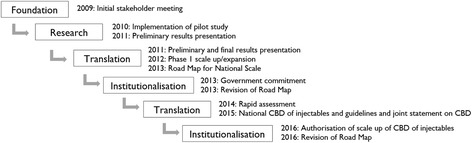
Timeline of research utilisation (RU) process for Zambia community-based access to depot-medroxyprogesterone acetate (DMPA)

In this case study, activities overlapped between the Translation and Institutionalisation phases, with considerable movement between the two (Fig. 2). Following the dissemination of preliminary findings, a larger dissemination meeting with more stakeholders was held in October 2011 to present the full pilot study results. Knowledge products included a Road Map for National Scale-Up of the programme which had been drafted based on the preliminary findings. At the October meeting, members of the General Nursing Council and the Health Professionals Council of Zambia were vocal about their concerns with lower level cadres administering injections. It was thus recommended that the MOH engage with these professional bodies to clarify issues and assuage their concerns. Representing an incremental decision to adopt, the MOH agreed to continue service delivery in the pilot districts without interruption and to revise the National Health Policy to allow provision of DMPA by CBD agents. All present at the meeting endorsed the Road Map for National Scale-Up. Soon after, government leadership changed, necessitating renewed advocacy targeting new MOH staff. Involving a highly respected and influential policy level champion as a knowledge broker throughout the translation phase supported the engagement of key government decision-makers, built their trust in the interpersonal and organisational processes, and enhanced the communication between all the key actors. The champion was a Zambian obstetrician and gynaecologist with extensive experience in FP and maternal health programmes at both the country and global level. He was familiar with FHI 360’s global work in expanding community-based access to injectables through community health workers, was a strong supporter of the practice, and felt passionate about advocating for policy-level changes in Zambia.

Initial expansion within the pilot study districts and one new district began in January 2012 and required technical assistance to the MOH and the newly formed Ministry of Community Development, Maternal and Child Health to advance the policy change dialogue and foster high-level ownership of the Road Map. By July 2012, the government pledged to increase contraceptive prevalence through various strategies, including reducing barriers to task sharing and doubling budget allocations for FP, an important step toward institutionalisation. In September 2012, a delegation of Zambian stakeholders participated in a study tour to observe Rwanda’s robust CBFP programme and to engage with stakeholders around important policy-level and operational issues. Despite ongoing stakeholder engagement and advocacy during this period, DMPA services stalled by June 2013 due to the lack of policy change and funds. However, high-level endorsements, including from the First Lady of Zambia, helped spur subsequent actions, including a rapid assessment of the performance of CBD distribution of DMPA and development of a National CBD of Injectables Strategy. Additional technical assistance was provided in the development of the guidelines, training curriculum and programme implementation tools based on learning from the pilot project. On January 22, 2016, the Zambia MOH gave official authorisation to scale-up the use of CBDs in the provision of injectable contraceptives. With funding, tools and guidance in place, and technical capacity within implementing organisations, this decision reflects a substantive move into the institutionalisation phase.

## Future applications in the real world

Ensuring that evidence is understood and meaningfully applied to programmes and polices requires intentional and systematic efforts. This simple, pragmatic framework provides a roadmap for the variety of inputs that are needed to undertake research with the end in mind, ensuring that key actors are engaged at various time points for the production and use of relevant evidence in programmes and policies. We have provided concrete examples of how RU activities within the framework phases were applied, drawing from a real-world case study of CBD of FP services in Zambia, just one of many experiences from over a decade of implementing global health research and programmes that helped to shape the development of this framework. In so doing, we aimed to add value over existing RU frameworks. Specifically, we posit that this framework:

- is simple enough for use by many different audiences, including end users of evidence;
- captures the fluid and dynamic RU process through its circular representation and explicit acknowledgement of feedback loops;
- is relevant for application in LMICs, having drawn from our organisational expertise working in these contexts;
- builds upon other evidence-based RU frameworks;
- highlights the important roles of, and interplay between, three categories of key actors, namely evidence producers, knowledge brokers and end users of evidence, all of whom must interact and collaborate for the success of RU efforts;
- focuses attention on the required inputs, including time and activities within the important translation phase;
- can be complimented by a suite of RU tools that we have developed.

Like any framework, this one has its limitations. As we have already noted, it is impossible to develop a framework that will fit every practitioner’s needs, particularly for such a dynamic and complicated process as RU. We opted to keep this framework simple and, in so doing, may not have represented all of the complex systems in which global health and development programmes must work. Nonetheless, it draws upon and reflects a thorough examination of the complex dynamics involved in achieving research utilisation. There may be additional expenses for implementing RU strategies and applying the framework, such as use of knowledge brokers, which are unavailable in non-grant funded situations. Furthermore, many external factors that may dictate the success or failure of research and evidence uptake, such as capacity or political climate, are not visually captured.

Like any process, it is important to evaluate whether the RU efforts actually result in increased uptake. Our framework emphasises building on evidence-based interventions, dynamic feedback and iterative processes; however, there is no visual evaluation component in the framework. While there is an increased recognition in the knowledge translation world of the need for evaluation of RU efforts, evaluation of these types of interventions is complex and assessing the impact of research uptake has often been limited to bibliometric methods [46], small sample surveys of researchers and decision-makers [47, 48], or qualitative methods to capture perspectives [18]. A mapping study of the knowledge mobilisation landscape in healthcare, social care and education found that formal evaluation of RU activities were rare [49]. Future research is needed on evaluation techniques for RU activities, particularly to assess their processes and impacts.

Application of this framework offers the potential to improve policy and programmes by increasing the likelihood that research results will be relevant, well-documented, easily communicated and understood, scalable, and built upon existing systems. We hope that global health and development stakeholders will apply it, document its use, assess its utility and improve upon it. It is not enough to just invest in research.

## Abbreviations

CBD: community-based distributors
CBFP: community-based family planning
DMPA: depot-medroxyprogesterone acetate
FP: family planning
LMIC: low- and middle-income country
MOH: Ministry of Health
RU: research utilisation
USAID: United States Agency for International Development

## Footnotes

1 FHI 360 is a non-profit human development organisation dedicated to improving lives in lasting ways by advancing integrated, locally driven solutions. FHI 360 serves more than 70 countries and all of the United States and its territories. More information can be found at: http://www.fhi360.org/

2 A package of research utilisation strategic planning tools are available at: https://www.fhi360.org/resource/research-utilization-strategic-planning-tools
